# Enovis SMR reverse shoulder arthroplasty for complex proximal humerus fractures in patients over 80 years of age

**DOI:** 10.1016/j.jsea.2026.100039

**Published:** 2026-05-27

**Authors:** Vittorio Candela, Yuri Piccolo, Lorenzo Giovinazzo, Daniele De Meo, Carmine Zoccali, Stefano Gumina

**Affiliations:** Department of Anatomy, Histology, Legal Medicine and Orthopedics, Sapienza, University of Rome- Policlinico Umberto I, Rome, Italy

**Keywords:** Reverse shoulder arthroplasty, Proximal humerus fracture, Tuberosity healing, Clinical outcomes, Internal rotation, Tuberosity fixation

## Abstract

**Background:**

Literature focusing on reverse shoulder arthroplasty (RSA) for complex proximal humerus fractures (PHFs) in patients aged ≥ 80 years is limited. This study aimed to evaluate clinical and radiological outcomes of RSA and tuberosity fixation for complex four-part PHFs in elderly patients, with a minimum follow-up (FU) of 24 months. The secondary aim was to assess tuberosities healing rate and correlate it with clinical outcomes.

**Materials and methods:**

We retrospectively analyzed 26 patients (8 males, 18 females; mean age 84.6 years) who underwent cementless RSA for four-part PHFs. At the final FU, Constant-Murley score (CS), a 3-grade satisfaction scale and VAS scale were recorded. Radiological healing of greater (GT) and lesser tuberosity (LT) was also evaluated.

**Results:**

At a mean FU of 38 months, the mean CS was 61.30 ± 5.61 (range 38-76). Tuberosity healing occurred in 88% of patients. No significant difference (*P* > 0.05) in CS was found between patients with GT healing (62.80 ± 5.30) and those without (59.70 ± 1.61). Active external rotation (ER) was significantly higher in patients with GT healing (22° ± 6.28) than without (4° ± 2.18). Patients with isolated GT healing showed greater ER and internal rotation (IR) than those with combined GT-LT healing in the combined group (27° ± 7.58 vs. 16° ± 3.28, *P* < 0.01). Patients satisfaction was high (95%). No complications, except for one acute periprosthetic infection occurred.

**Conclusions:**

RSA is a safe and effective treatment for complex four-part PHFs in patients over 80 years. The high tuberosity healing rate (88%) underlines the importance of fixation technique; however, tuberosity healing did not correlate with overall shoulder function but only with active external rotation. As for patients around 70 yrs, healing of LT was linked to GT healing, as isolated LT healing was never observed.

A recent epidemiological study on a large sample demonstrated that more than 60% of all 4-part proximal humerus fractures (PHFs) occur in individuals aged 80 years or older.^27^ Considering the high degree of osteoporosis and the risk of avascular necrosis, these fractures, when surgery is indicated due to the patient's general condition and activity level, require a reverse shoulder arthroplasty (RSA).^1^^,^^3^^,^^4^^,^^7^^,^^26^ Good to excellent clinical outcomes with a low complication rate have been reported,^14^^,^^21^ independently by how tuberosities were managed.^5^^,^^8^^,^^11^^,^^25^^,^^28^^,^^32^ However, looking deeply the recent literature, patient's reported age is around 70 years,^6^^,^^12^^,^^16^^,^^20^^,^^22^^,^^26^^,^^29^^,^^31^ and also in case of studies that included patients aged over 80 years,^2^^,^^30^^,^^33^ clinical outcomes data and complication rate were fractioned requiring extrapolation and making samples less representative. Furthermore, no data are available regarding the potential healing rate of tuberosities after RSA in very elderly population.

Considering these premises, our aims were to evaluate both clinical and radiological outcomes in patients over 80 years old treated with RSA with tuberosity fixation for complex four-part PHFs, after a minimum follow-up (FU) of 24 months. The secondary aim was to evaluate the healing rate of the greater tuberosity (GT) and lesser tuberosity (LT) and correlate it with clinical outcomes.

## Materials and methods

Thirty-five patients older than 80 years who underwent cementless RSA (SMR Shoulder, Enovis) from January 2015 to June 2023 for four-part PHFs/fracture dislocation were retrospectively enrolled.

For all patients, preoperative evaluation of the fracture pattern was performed with standard trauma-series X-rays and a computed tomography (CT) scan. Fractures were classified according to Codman Lego system and the risk of avascular necrosis was calculated according to Hertel predictor criteria.^19^ However, the final decision to implant a RSA was made intraoperatively. Patients posture types were categorized according to Moroder et al^23^ classification.

Exclusion criteria were applied as follows: pathologic fractures of neoplastic origin, fractures with associated neurologic lesions, patients with associated fractures in other bone segments, and patients with chronic inflammatory diseases.

All surgeries were performed at the same hospital, with patients in the beach-chair position under general anesthesia and interscalene block. A deltopectoral approach was used in all cases. The surgical technique was the same as that described by Candela et al^8^ Glenosphere of 36 mm, 40 mm, and 44 mm were implanted in 10, 19, and 6 patients, respectively. In 28 and 7 patients, a SMR glenoid baseplate and a 7° superior augmented baseplates were implanted, respectively. Standard and mini press-fit stems were used according to the extension of the fracture line distal to the surgical neck. Reverse humeral bodied were implanted with 10° (posture type II) or 20° (posture type III) of retroversion using proper instrumentation aligned to the forearm according to posture types.^23^ Tuberosities were fixed using a previously described interposition graft technique.^8^

Postoperative protocol: all patients followed the same postoperative protocol, which consisted of three weeks of immobilization with a sling in a neutral rotation and 15° of abduction. Passive and active-assisted shoulder range of motion were allowed starting on the day of sling removal, with daily sessions conducted by an experienced physiotherapist, in addition to a self-managed rehabilitation program.

Clinical and radiological FU anteroposterior and true axillary radiographs were obtained at 1, 3, 6, 12, and 24 months postoperatively, and at the final FU visit. The GT and LT were considered as healed when consolidated to the humeral diaphysis in the AP and in the true axillary view.^25^ The evaluation was performed by an experienced radiologist blinded to the study purpose and repeated 3 times in different moments. Constant Murley score (CS) was calculated at the final FU. Patient's satisfaction was recorded using a 3-grade scale (very satisfied, satisfied, and disappointed). Early (<30 days) and late complications were documented.

### Power analysis

Sample size calculation was performed using G∗Power 3.1.9.4 software (Heinrich-Heine-University, Dusseldorf, Germany). According to the a priori power analysis, at least 18 patients would be required to get a 25% better GT healing, assuming a 2-tailed a-value of 0.05 (sensitivity of 95%), and ab value of 0.95 (with a study power of 95%).

### Statistical analysis

Kolmogorov-Smirnov test was used to assess the normal data distribution. Categorical variables were calculated using frequencies and proportions whilst continuous data were estimated by means, standard deviations and ranges. Differences between 3 groups for all data have been analyzed using one-way ANOVA test. Significant levels for multiple comparisons were adjusted using the Bonferroni procedure. Mann-Whitney *U* test has been used to analyze differences between 2 groups. In addition, Kruskal-Wallis test was applied to nonparametric ordinal data for multiple groups. The Statistical Package for Social Sciences (version 25; IBM Corporation, Armonk, NY, USA) was used for calculations and data were analyzed by a single researcher. Calculated *P* values were 2-sided, *P* < .05 was considered statistically significant, and all results are reported with a 95% confidence interval where appropriate.

## Results

**Figure 1:**
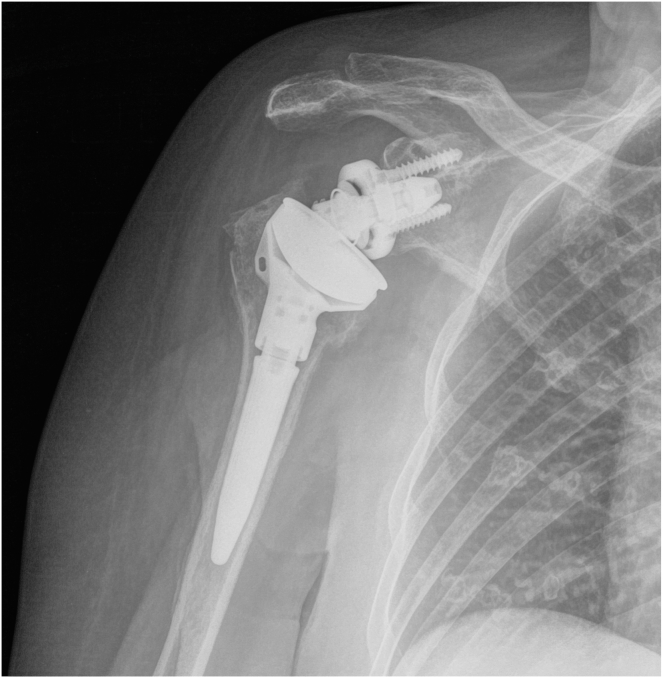
*Right* shoulder follow-up radiograph at 31 months postoperatively (83 year old), showing proper healing of both the LT and GT. *LT*, lesser tuberosity; *GT*, greater tuberosity.

**Figure 2:**
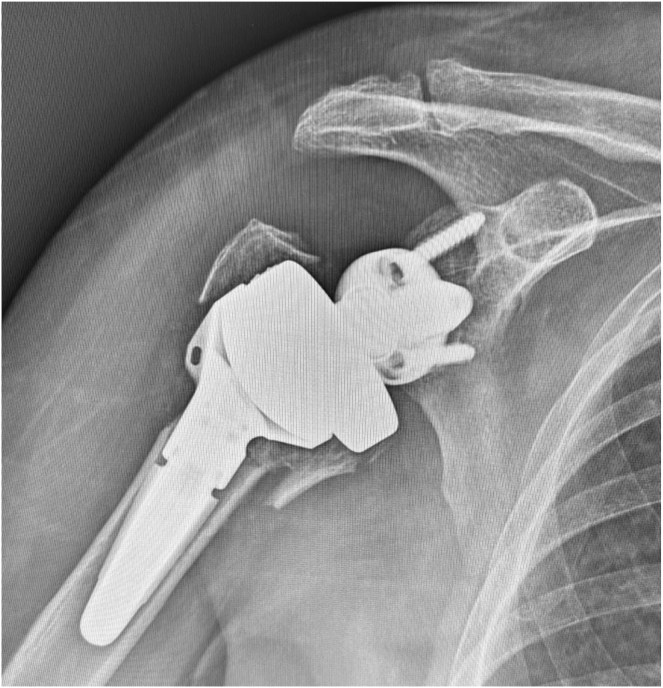
Follow-up radiograph of a *Right* shoulder (86 year old) at 12 months postoperatively, showing nonunion of the tuberosities with migration.

**Figure 3:**
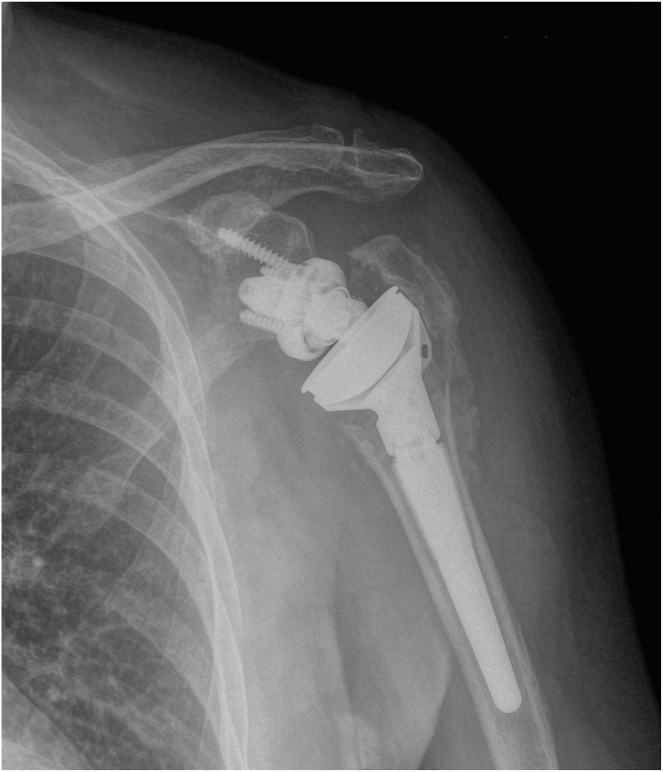
Follow-up radiograph of *Left* shoulder at 24 months postoperatively. In this 82-year-old patient, a malunion of the GT is observed without any migration. *GT*, greater tuberosity.

The final study group comprised 26 patients (8 males – 18 females; mean age, 84.6 years, range: 80-88 years). The mean FU was 38 months (range 24-96 months). The following nine patients were excluded: 3 with rheumatoid arthritis, 2 with an ipsilateral olecranon fracture, 1 with a bilateral humeral fracture, and 2 with preoperative ulnar nerve palsy and 1 additional patient was lost to FU. The average time from injury to surgery was 9 days (range 2-14 days). The dominant side was involved in 18 of 26 cases. In 17 of 26 cases, a Hertel type 12 fracture was treated, while 9 patients had a fracture-dislocation of the proximal humerus. According to Scapular orientation and posture classification,^23^ type B and C were identified in 11 patients and 15 patients, respectively. Mean surgery time was 69 minutes (range 50-102 minutes). No patient required blood transfusion. Regarding the radiological evaluation a high level of intra-rater reliability was observed, with an intraclass correlation coefficient of 0.89 (95% confidence interval: 0.82–0.94); tuberosity healing was found at the final FU in 23 of 26 cases (88%) (Fig. 1). Nonunion was observed in 2 patients (81-year-old male and 83-year-old female), and GT migration was documented in 1 case (82-year-old female) (Figs. 2 and 3). When the GT healed, the LT healed in 20 of 23 cases. Isolated healing of the LT was never observed. The mean CS was 61.30 ± 5.61 (range 38-76), with subscores as follows: pain, 14 ± 1 (range 6-15); activity, 16 ± 3 (range 7-19); mobility, 25 ± 5 (range 12-38); and strength, 6 ± 5 (range 0-16). When stratifying by GT healing, no clinical difference was observed in the CS; mean CS in GT healing group was as follows: 62.80 ± 5.30; mean CS in nonhealing group was as follows: 59.70 ± 1.61). Active external rotation (ER) was greater in patients with GT healing (22° ± 6.28) compared with those with GT resorption or migration (4° ± 2.18). No differences were found regarding the mean forward flexion (healed GT: 143° ± 18° vs. GT resorption: 137° ± 13°). Patients with isolated GT healing showed greater external and internal rotation (IR) compared to those with combined GT and LT healing: mean ER in the isolated GT group was 27° ± 7.58, compared to 16° ± 3.28 in the combined GT-LT group, mean IR in the GT group was L1 vs. L4 for GT-LT group. At the final FU, 17 (65%) and 8 (30%) patients were very satisfied and satisfied, respectively. The patient with GT migration was not satisfied with the clinical outcomes.

One complication occurred: an acute periprosthetic infection, treated within 2 weeks of the onset of symptoms with debridement, antibiotic pearls, irrigation and implant retention, resuming the rehabilitation process normally.

## Discussion

The present study analyzed clinical and radiological outcomes of a cohort of patients older than 80 years submitted to RSA for complex four-part PHFs. Our findings suggest that RSA is a safe and satisfactory procedure for treating complex fractures in an active elderly population. The sample included patients with four-part fractures and four-part fracture-dislocation of the proximal humerus. Complex three-part PHFs associated with low risk of avascular necrosis were excluded because in our daily practice excellent clinical and radiological outcomes after open reduction and fixation, with a construct of blocked threaded wires, in elderly active patients have already been reported.^10^^,^^17^^,^^18^ When the fracture was associated with a high risk of avascular necrosis (four-part proximal humeral fractures/fracture dislocation) and the patient was considered active based on lifestyle (patient autonomous in common activities of daily living and in personal hygiene care), a RSA was implanted.

All patients received the same RSA implant with a dedicated fracture stem; humeral version was adjusted considering scapular orientation, in accordance with studies demonstrating improved outcomes when version is personalized based on posture.^23^^,^^24^ During the FU, no complication occurred except for an acute periprosthetic infection treated with implant retention; no intraoperative or postoperative periprosthetic fractures, no dislocations were reported and these data confirm RSA safety also in the elderly population. RSA efficacy was confirmed by the mean CS was comparable to those of previous published papers^8^^,^^25^^,^^28^ involving younger patients undergoing RSA for complex PHFs. The true functional impact of tuberosity fixation and healing in RSA remains a debated topic. Jonsson et al^20^ reported no significant differences in shoulder function between patients with healed tuberosities versus those with failed healing. Similarly, Torrens et al^32^ and Porcellini et al^28^ found no notable differences in terms of CS between patients with healed and unhealed GTs. Furthermore, Grassi and Zorzolo^15^ noted no correlation between the radiographic appearance of GT and clinical outcomes, concluding that neither tuberosity healing necessarily lead to higher CS results nor did resorption result in functional impairment. In our sample, tuberosity fixation was performed with the interposition graft technique.^8^ In our opinion, fixation of tuberosities is essential to achieve the correct deltoid wrapping and the implant stability, without excessive distalization of the center of rotation or conjoined tendon tension.^9^^,^^14^ Tuberosity healing was observed in 88% of cases, despite the advanced age of the patients. Surprisingly, this rate is higher than the one reported in a previously published series composed of younger patients submitted to the same RSA implant for complex four-part PHFs.^8^ This may be explained by the fact that healing depends more on correct tension and height, achieved through the use of an interposition autologous graft, and proper contact with the humeral shaft, as demonstrated by Ohl et al^25^ and confirmed recently by Porcellini et al,^28^ with respect of patient's age. No significant differences in CS were found between patients with and without tuberosity healing, which is similar to previously published papers.^8^^,^^13^^,^^15^^,^^20^^,^^28^ However, considering only range of motion, patients with healed GT or GT-LT showed significantly greater active ER compared to those without healing. This result was expected and previously documented in younger population and valid also for the elderly patients.^5^^,^^14^

Our findings seem to align with the latter perspective. Specifically, in the GT-LT healed patients, the subscapularis fixed in a neutral position may restrict the active ER that could be produced by an aging posterior infraspinatus/teres minor. Despite this, it is technically demanding to achieve a stable GT fixation excluding the complex LT-subscapularis, so our surgical technique continues to include the fixation of both tuberosities. Furthermore, patients with GT-LT healed showed lesser IR with respect to those with only GT healed. It could be justified by the possible anterior impingement between the LT and the conjoint tendon complex.^9^

This study has several limitations. First, it has a retrospective design. Second, only radiographs were used to evaluate tuberosity healing, with no CT scans performed. However, given the absence of complications, the use of CT imaging was not ethically justified considering also the very elderly population. Lastly, the FU period was medium-term, and longer-term radiological results remain to be assessed.

## Conclusion

RSA is a safe and effective treatment for complex four-part PHFs in patients over 80 years, yielding satisfactory functional outcomes, a low complication rate, and high patient satisfaction. The healing rate of tuberosities was high (88%) despite patients age, demonstrating that the fixation technique is the key to achieve healing and patient satisfaction; however, tuberosities healing did not correlate with overall shoulder function.

## Disclaimers:

Funding: No funding was disclosed by the authors.

Conflicts of interest: The author, their immediate family, and any research foundation with which they are affiliated have not received any financial payments or other benefits from any commercial entity related to the subject of this article.

Patient consent: Obtained.
